# Optical multi-channel interrogation instrument for bacterial colony characterization

**DOI:** 10.1371/journal.pone.0247721

**Published:** 2021-02-25

**Authors:** Iyll-Joon Doh, Huisung Kim, Jennifer Sturgis, Bartek Rajwa, J. Paul Robinson, Euiwon Bae

**Affiliations:** 1 School of Mechanical Engineering, Purdue University, West Lafayette, Indiana, United States of America; 2 Basic Medical Sciences, College of Veterinary Medicine, Purdue University, West Lafayette, Indiana, United States of America; 3 Bindley Bioscience Center, Purdue University, West Lafayette, Indiana, United States of America; 4 Weldon School of Biomedical Engineering, Purdue University, West Lafayette, Indiana, United States of America; Texas A&M University, UNITED STATES

## Abstract

A single instrument that includes multiple optical channels was developed to simultaneously measure various optical and associated biophysical characteristics of a bacterial colony. The multi-channel device can provide five distinct optical features without the need to transfer the sample to multiple locations or instruments. The available measurement channels are bright-field light microscopy, 3-D colony-morphology map, 2-D spatial optical-density distribution, spectral forward-scattering pattern, and spectral optical density. The series of multiple morphological interrogations is beneficial in understanding the bio-optical features of a bacterial colony and the correlations among them, resulting in an enhanced power of phenotypic bacterial discrimination. To enable a one-shot interrogation, a confocal laser scanning module was built as an add-on to an upright microscope. Three different-wavelength diode lasers were used for the spectral analysis, and high-speed pin photodiodes and CMOS sensors were utilized as detectors to measure the spectral OD and light-scatter pattern. The proposed instrument and algorithms were evaluated with four bacterial genera, *Escherichia coli*, *Listeria innocua*, *Salmonella* Typhimurium, and *Staphylococcus aureus*; their resulting data provided a more complete picture of the optical characterization of bacterial colonies.

## 1. Introduction

Bacterial colonies consist of millions of cells of the bacteria, and extracellular matrices that are secreted from cells render bacterial colonies into a complex 3-D structure. In essence, both microscopic and macroscopic characteristics emerge from the 3-D structural properties. Interrogation and characterization of colonies are challenging owing to the size differences and the aggregation of smaller cells. Optical microscopy techniques are traditionally adopted to analyze the physical characteristics of the bacterial colonies. However, owing to common morphological similarities among colonies formed by different organisms, these conventional methods have difficulties in performing bacterial classification. One alternative method of characterizing bacterial colonies is elastic light-scattering (ELS) technology, which interrogates the whole 3-D volume of a colony using a collimated and coherent light source and projects the interference patterns into 2-D scattering images [1]. As the unique scatter patterns are generated depending on the colony structure, classification of a bacterial sample has been successfully performed for different genera, species, and some level of strains in different conditions [2]. For its simple and rapid detection methods, the optical technique has gained interest and attention, which have been expressed in various studies where similar approaches were applied for bacterial colony characterization [3–6].

According to the scalar diffraction theory, the light-scatter pattern resulting from the interaction between the incoming laser beam and the whole volume of the bacterial colony depends on the colony morphology and its constituents. This fact implies that the colony’s morphological characteristics and relevant growth properties are essential in interpreting the light-scatter pattern generation. Therefore, studies have been carried out to investigate the correlation between the light-scatter pattern and colony morphology, characterized as color, size, shape, texture, opacity, etc [7–10]. However, these studies require multiple measurements by different instruments, requiring samples to be transferred to different stations equipped with various instrumentation. Since bacterial colonies are continually growing, either different colonies have to be measured simultaneously using various instrumentations or multiple measurements on a single colony must be made serially over a significant amount of time. To cope with this situation and overcome the limitations of the previous design, we propose combining multiple microscopic modalities with the ELS technique, as multi-channel instruments are commonly found in various fields and provide applicability, versatility, and improved efficiency over a single modality device [11,12].

**Fig 1:**
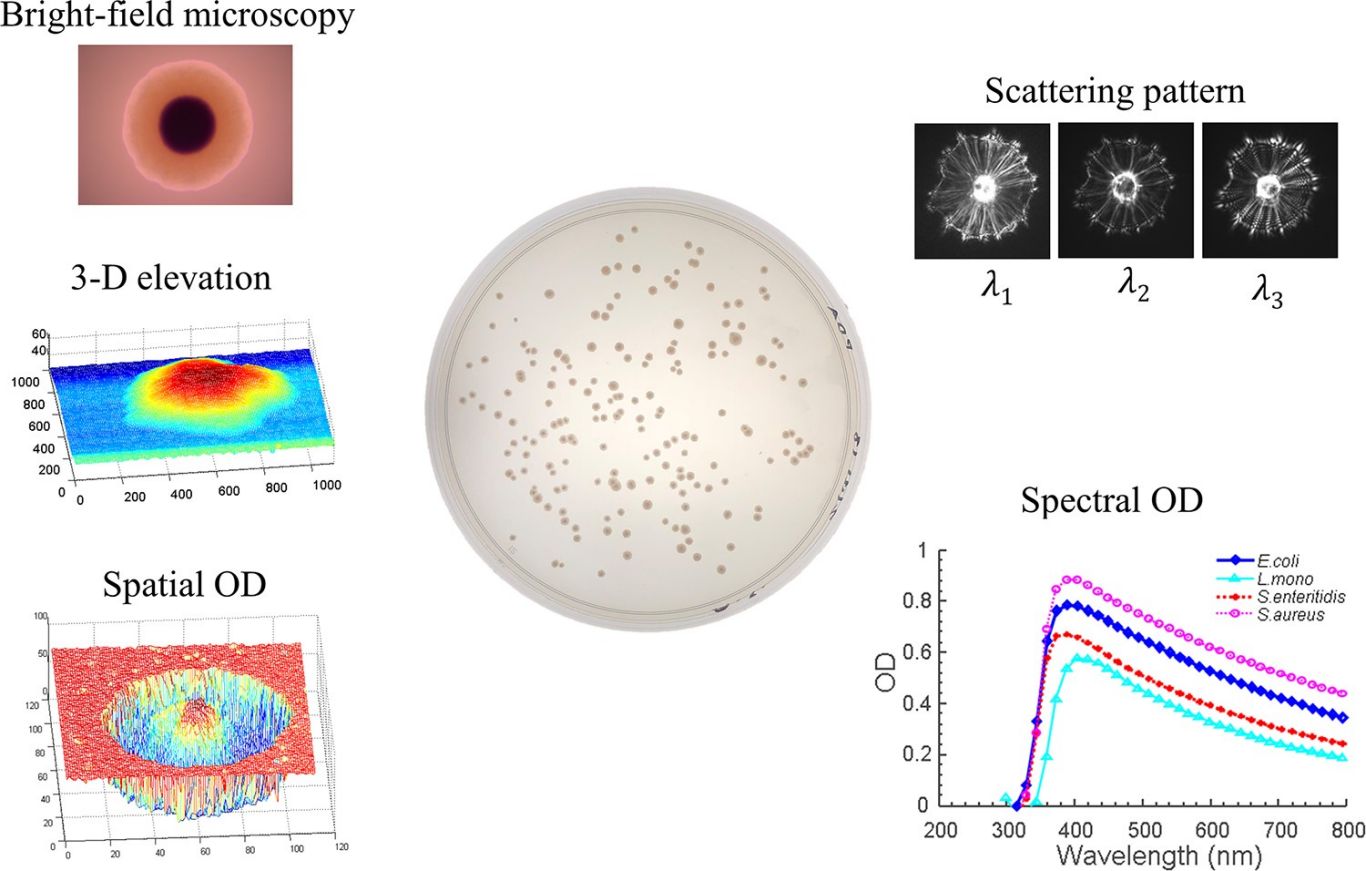
Graphic illustration of the available channels. Proposed measurement methods of the multi-channel bacterial colony analyzer: Bright-field microscopy, 3-D morphology map, spatial optical-density distribution, spectral light-scatter patterns, and spectral optical density.

This paper presents a new instrument concept that can deliver five different optical channels to interrogate bacteria colonies. The new instrument can offer 1) bright-field light microscopy, 2) 3-D colony morphology maps, 3) 2-D spatial optical density (OD) maps, 4) spectral forward-scattering pattern, and 5) spectral optical density for bacterial colonies (see Fig 1). This multi-channel instrument can measure a series of bio-optical characteristics without moving the sample; this approach can enhance our understanding of the correlation between colony morphology and light-scatter patterns and potentially improve the bacterial classification. The instrument was designed with a commercial infinity-corrected microscope on which a custom-built confocal module, three laser diodes, and optical sensors were installed. Colonies from four major bacterial genera, *Escherichia coli*, *Listeria innocua*, *Salmonella* Typhimurium, and *Staphylococcus aureus*, were tested. The performance of each optical modality was evaluated, and the morphological and optical properties of the bacterial colony that are correlated to the light-scatter pattern were thoroughly investigated.

## 2. Materials and methods

### Instrument design

**Fig 2:**
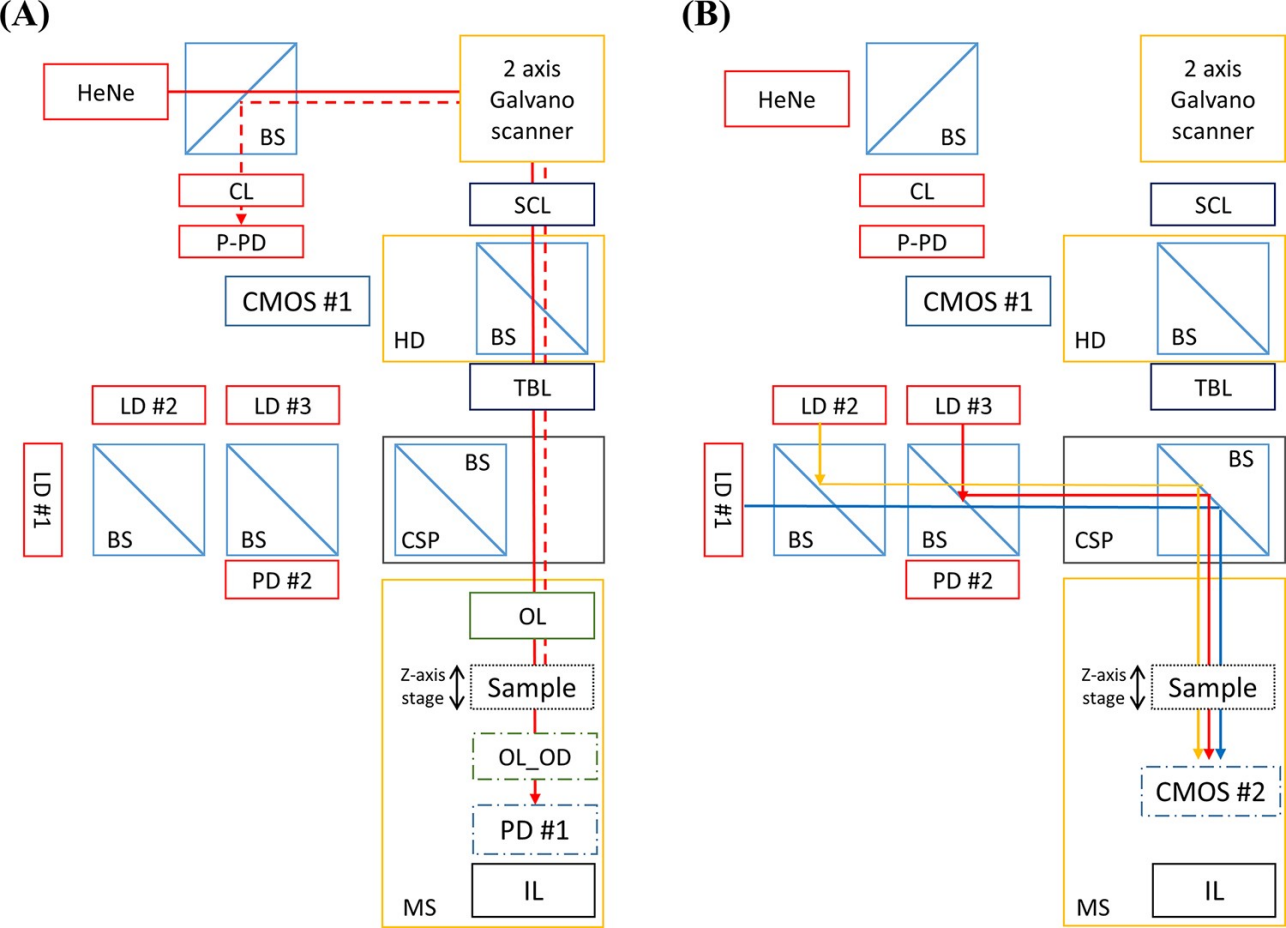
Schematic and operation diagram of the multi-channel instrument. Illustration of the schematic of (A) confocal and (B) spectral ELS modes of the instrument. Components represented are He-Ne laser (HeNe), 2-axis galvanometer, laser diode (LD), photodiode (PD), beam splitter (BS), collimator (CL), pigtailed pin PD (P-PD), CMOS sensor (CMOS), scan lens (SCL), tube lens (TBL), microscope body (MS), microscope head (HD), custom-built panel for beam splitter (CSP), objective lens (OL), objective lens for optical-density measurement (OL_OD), and illumination (IL). Solid lines represent the optical path towards and through the sample, dashed lines the optical path of light reflected from the sample.

An overview and schematic diagrams are illustrated in Fig 2, including the components of the proposed instrument. An infinity-conjugated microscope (BX51, Olympus Corp., Tokyo, Japan) was utilized as the base frame of the instrument to ensure mechanical stability, compatibility, and further expandability. For the bright-field light microscopy, an image sensor (CMOS #1) (PL-B471, Pixelink, Ottowa, ON, Canada) was mounted at the eyepiece to capture magnified images of bacterial colonies. The 3-D colony morphology and spatial OD maps were measured by scanning a sample colony using a custom-built confocal laser scanning module attached to the microscope; the module consists of a collimated He-Ne laser (05-LHP-991, Melles Griot, Carlsbad, CA, USA), a 2-axis mirror galvanometer (GVS201, Thorlabs Inc., Newton, NJ, USA), two high-speed pin photodiodes (S5971, Hamamatsu Photonics, Shinjuku City, Tokyo, Japan), and various optical lenses and beamsplitters. One of the pin photodiodes (P-PD) was pigtailed to an optical fiber (SM600, Thorlabs Inc., Newton, NJ, USA), and the other end of the fiber was connected to a collimating lens (CL) to collect the reflectance signal from the sample for the 3-D colony-morphology map. The other photodiode (PD #1) was placed under the sample plate to measure the transmitted light for the spatial OD map. Pictures of the proposed instrument are shown in S1 Fig.

To control the mirror galvanometer and process the reflectance and transmittance signals, a dual-core MCU (TMS320F28377S, Texas Instruments, Dallas, TX, USA) with 200-MHz clock speed was selected for the main processor. The mirrors’ position was controlled and monitored through 12-bit DAC and ADC channels, and the collected signals were converted to digital data through other 12-bit ADC channels. A multi-purpose pre-amp circuit board was designed, including a current-voltage converter with a voltage reference unit, two inverting amplifiers, and a 2^nd^-order active filter (see S2 Fig). With a simple wiring modification, the circuit board was used as a pre-amp for high-speed PDs and a signal compensator for the galvanometer. The main timer interrupt was running at 16 kHz for a raster-scanning motion, while X and Y-axis mirrors were moving at 128 Hz and 1 Hz, respectively. Therefore, 128 × 128 scanning was completed in a second. All sequences were controlled by a custom-built XML-based GUI developed in MS Visual Studio. A 3-D visualizing library from MATLAB was cross-compiled as a 3-D image viewer in the GUI.

A custom beamsplitter panel (CSP) was installed on the microscope to switch between microscope mode and spectral ELS mode. To create the spectral scattering patterns, three laser diodes (LD#1, LD#2, and LD#3) with wavelengths of 405 (Excelitas Technologies Corp., Waltham, MA, USA), 635 (Coherent 0221-698-01 REV B, Coherent Inc., Santa Clara, CA, USA), and 904 nm (Lasermate, Walnut, CA, USA) were selected based on the wavelengths selected in the previous report [13]. The individual wavelength was selected from the commercially available wavelength of the laser diode, and the separation between wavelengths was maximized to observe the clear effect of the wavelength. Another pin photodiode (PD #2) was placed along with the three lasers to measure the input power of the lasers for spectral OD measurement. The collimated beams were aligned to illuminate the same spot on the sample for simultaneous pattern generation utilizing pellicle beam splitters. To capture the patterns, a secondary CMOS sensor (CMOS #2) was placed below the sample plate, which was an interchangeable component for PD #1 for the spectral OD measurement.

### Sample preparation

For bacterial samples, *Escherichia coli*, *Listeria innocua*, *Salmonella* Typhimurium, and *Staphylococcus aureus* were prepared and tested on the proposed instrument. All cultures were first taken from a freezer at -80°C and streaked on one of the most common nutrient media, trypticase soy agar (TSA) (Bacto™, BD Diagnostics, Franklin Lakes, NJ, USA). The volume of the nutrient medium was fixed to 25 ml to maintain the same thickness of the agar. The streak plates were then incubated at 37°C until the colonies were visible. One colony from each genus was picked and serially diluted in 4 ml buffer solution (PBS) three times by a factor of 1:40. A 50-μl aliquot of the last dilution tube was spread on TSA and incubated at 37°C until the diameter of the colony reached an appropriate size for interrogation. The diameter of the colonies was controlled depending on the field of view (FOV) of the utilizing objective lenses. In this paper, the diameter of the colony was 400 to 600 μm for 10× and 600 to 1000 μm for 20× objective lenses.

### Operational procedure

The bright-field microscope images of a bacterial colony were measured at the eyepiece with CMOS #1. When the instrument was in microscope mode, the confocal module was enabled by preparing the objective lenses (OL and OL_OD) and PD#1 for the 3-D morphology and spatial OD measurements. As shown in Fig 2A, the two-axis galvanometer deflected the incoming laser beam from the He-Ne laser for raster scanning, and the OL focused the light source towards the sample colony. The reflected light from the focus point at the sample returned through the same path and focused on P-PD. Simultaneously, the transmitted light was collected by OL_OD and focused on PD #1, and the intensity was recorded for the spatial OD distribution. The 3-D image of the colony was acquired by stacking thin slices of confocal images, taken every 5 μm in the z-direction.

As illustrated in Fig 2B, the spectral ELS mode was enabled by removing OL and switching CSP for the three laser diodes. For the spectral OD measurement, each laser illuminated the sample one by one, and the intensity of transmitted light was measured with PD #1. The input intensity *I*_0_, a reference to the transmitted light *I*, was measured with PD #2 and used in Eq 1 to calculate the spectral OD, *Z_OD_*. For spectral scattering-pattern measurement, the sensor unit (OL_OD and PD#1 assembly) under the sample stage was replaced with CMOS #2. Similarly, the sample was illuminated by each laser, and the scattering pattern was captured by CMOS #2.

$$Z_{OD}=\log_{10}(I_{0}/I)$$ (Eq 1)

## 3. Results

### Overview of colony interrogation using the multi-channel instrument

**Fig 3:**
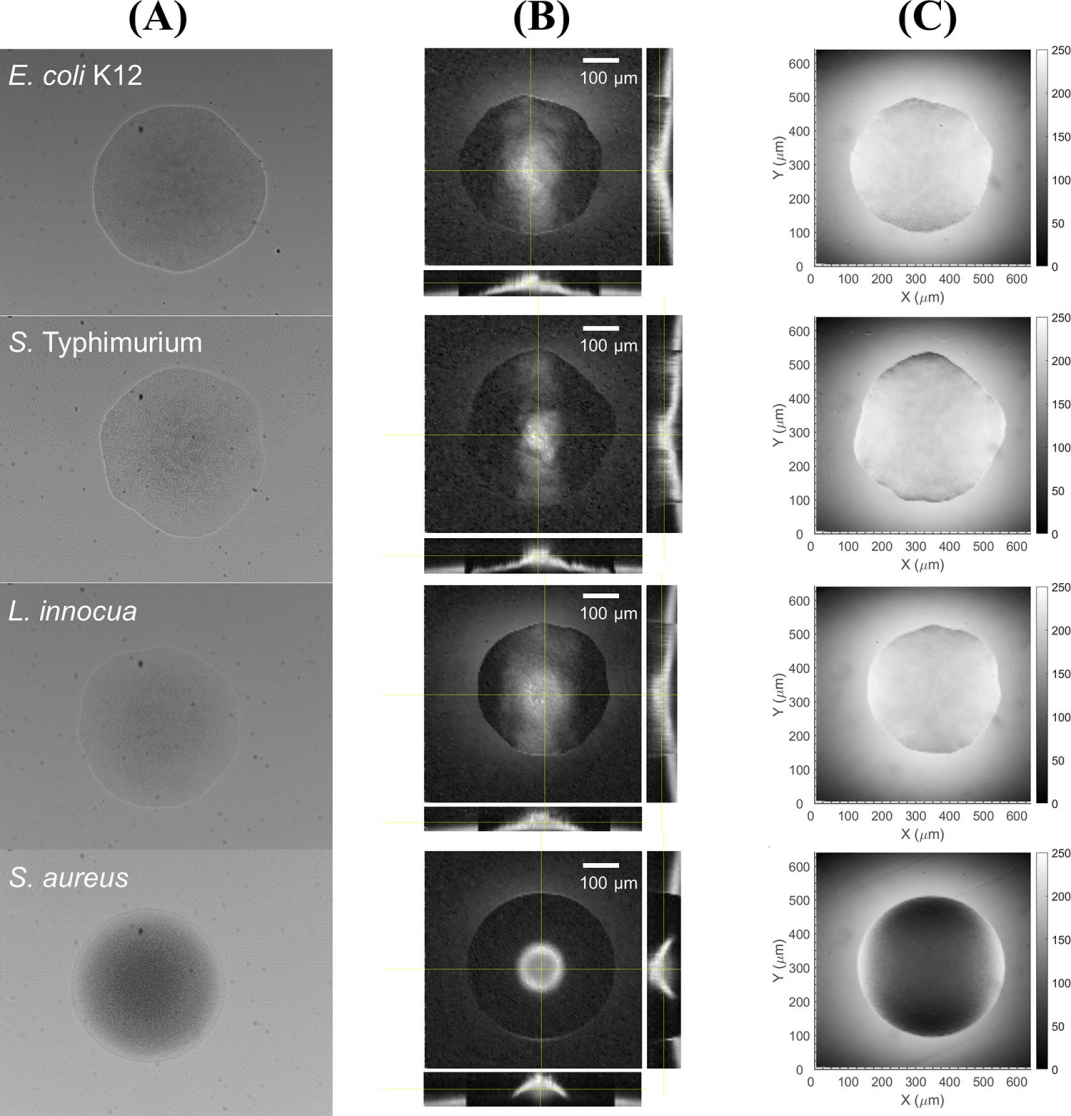
Microscopic analysis of sample bacterial colonies. Representative images of (A) bright-field microscopy, (B) 3-D morphology map, and (C) 2-D spatial OD map of *E*. *coli*, *S*. *Typhimurium*, *L*. *innocua*, and *S*. *aureus* colonies, measured with a 20× objective lens.

Utilizing the multi-channel bacteria colony analyzer, ten colonies per genera were interrogated with the five optical modalities without moving the specimen. The microscopic analysis was performed under microscope mode using a 20× objective lens with an NA of 0.5 (UPlanFl 20×, Olympus Corp., Tokyo, Japan), and their representative images are presented in Fig 3. The bright-field microscope images (Fig 3A) provided general insights into colony shape and opacity, and the stacked images of confocal microscopy (Fig 3B), referred to as a 3-D colony morphology map, offered additional information on the elevation profile as the cross-sections across the center of a colony in the vertical and horizontal directions were given together. Meanwhile, the spatial OD distribution map (Fig 3C) represented the intensity of the light transmitted through the colony, which varies depending on the colony profile and internal cell structure [14]. The qualitative comparison revealed that colonies of *E*. *coli*, *S*. Typhimurium, and *L*. *innocua* shared similarities in their shape, as their colonies had irregular forms and raised elevations with gentle slopes. Since they were still in the earlier stage of growth where the colony diameters were only 400–600 μm, distinctive morphological characteristics were not yet expressed, and it was difficult to distinguish one from another using only microscopic analysis. However, *S*. *aureus* still showed unique attributes in all three microscopic channels in that it had a perfectly circular form and high elevation with a steeper slope in the colony profile. Therefore, in Fig 3B, owing to the steeper slope of the *S*. *aureus* colony profile, the reflectance from the colony surface was not fully captured with the given numerical aperture of the mounted objective lens; as a result, the outer part of the colony was not clearly illustrated in the 3-D morphology map. *S*. *aureus* colonies were also much more optically dense than those of the other bacteria, resulting in opaque colony images in both bright-field microscope and spatial OD map. The average pixel intensities of the spatial OD distributions, compared in S3 Fig, also demonstrated that *S*. *aureus* colonies had the lowest average pixel intensities, indicating *S*. *aureus* had the colonies with highest ODs among the four bacteria.

**Fig 4:**
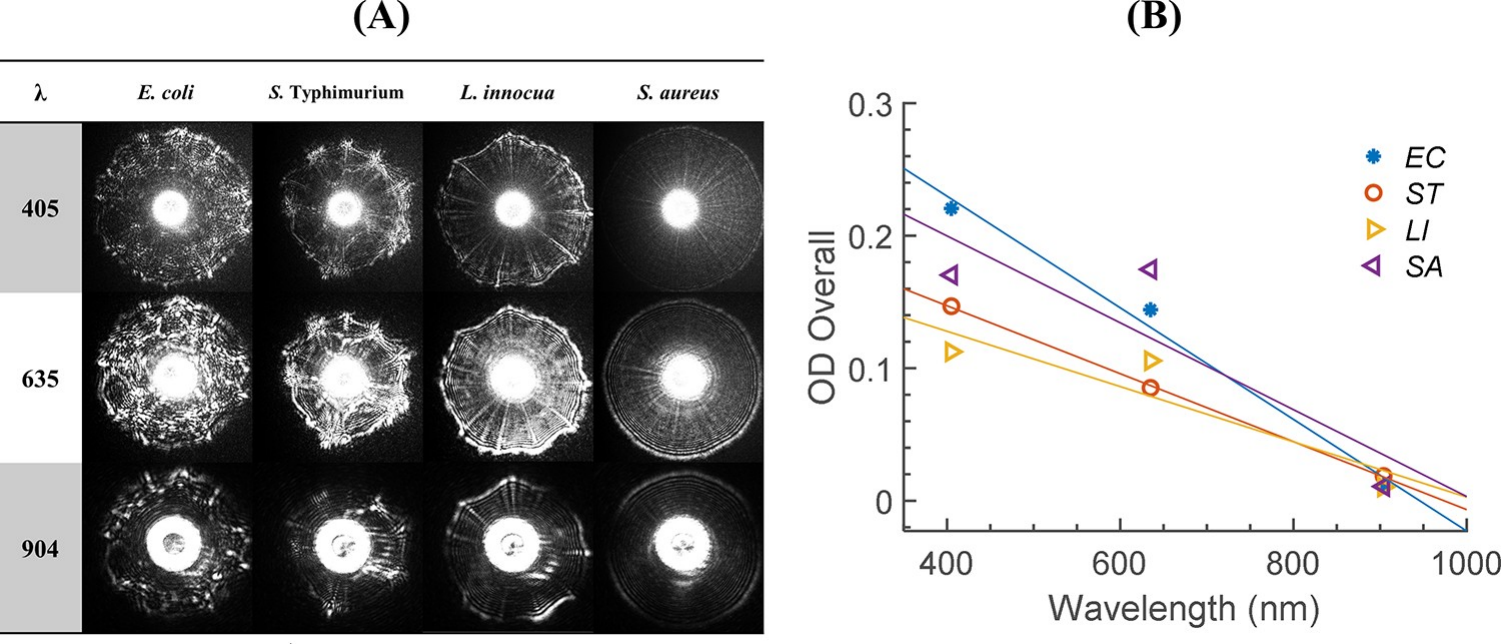
Spectral analysis of sample bacterial colonies. For spectral analysis, 405, 635, and 904 nm were utilized. (A) representative spectral light-scatter patterns and (B) spectral ODs for *E*. *coli*, *S*. *Typhimurium*, *L*. *innocua*, and *S*. *aureus* colonies. Each data point in (B) is an average of ten measurements (n = 10). The least-square fit was performed, and the regression lines are illustrated together.

Along with the microscopic analysis, the bacterial colonies were interrogated by three different wavelengths, 405, 635, 904 nm, to measure the spectral light-scatter patterns and spectral ODs. Representative scattering patterns of the four bacteria are presented for qualitative comparison in Fig 4A. Unlike the microscopic images, the scattering patterns showed apparent distinctiveness among the four bacteria genera as their unique morphological characteristics were amplified through the ELS phenomenon. The scattering patterns for *E*. *coli*, *S*. Typhimurium, and *L*. *innocua* had web-like patterns with spikes and radial spokes, which showed no uniformity induced from the uneven surface roughness and internal cell structure of the colony [9]. Nonetheless, the differences among the genera were evident as the shapes were uniquely arranged. Meanwhile, *S*. *aureus* colonies produced perfectly circular and concentric patterns that were clearly distinct from those of the other organisms. Not only the shape but also the pattern size was exceptional for *S*. *aureus* colonies; their patterns were much greater than the others and did not fit into the active area of the CMOS sensor. Thus, *S*. *aureus* was considered as an exception, and the image sensor was moved closer to the sample to fully capture the scattering pattern. In addition to the unique morphological characteristics embedded in the light-scatter pattern, the spectral scattering patterns provided wavelength-dependent features that enhanced the performance of bacterial classification [13,15]. In terms of pattern shape, the interference pattern such as rings and spokes became thicker, and the number of rings was decreased when the wavelength was increasing (See S4 Fig). Fig 4B shows spectral ODs of the four bacteria, and their linear regression lines were computed using spectral intensities. In general, all the sample bacteria output the lowest ODs at 904 nm, but the separation became apparent as wavelength decreased. At 635 nm, *S*. *aureus* colonies had the highest OD, *S*. Typhimurium the lowest. However, this tendency was changed at 405 nm, where *E*. *coli* and *L*. *innocua* resulted in the highest and lowest ODs, respectively. In contrast to the single-wavelength method, which provided limited differentiability among genera, ODs determined at three wavelengths can be utilized as a simple classification method at the genus level since the change of OD with respect to the wavelength could be a unique signature.

### Performance of the custom-built confocal module

**Fig 5:**
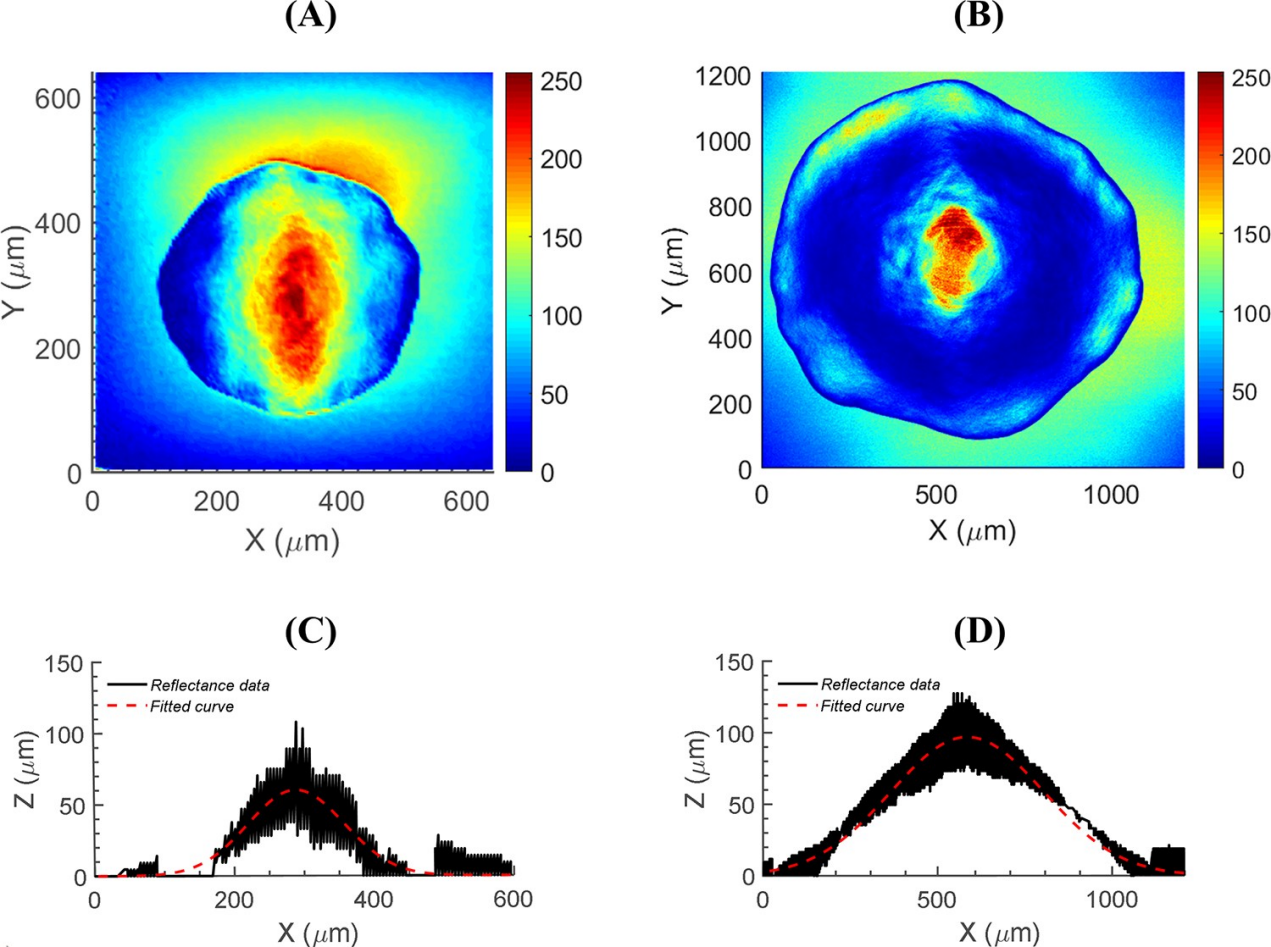
Performance evaluation of the custom-built confocal module. 3-D morphology maps of *E*. *coli* colony were measured by (A) multi-channel instrument using the custom-built confocal module, and (B) Bio-Rad Radiance 2100MO confocal microscope. The corresponding cross-section profiles at the center of the colony are given below as (C) and (D).

Owing to the confocal imaging system, the light-scatter patterns were collected together with the other valuable morphological information of the corresponding colony. To evaluate the performance of the custom-built confocal module, the instrument was examined against a commercial confocal imaging system (Radiance 2100MO, Bio-Rad Laboratories, Inc., Hercules, CA) mounted on an inverted microscope (Nikon Eclipse TE2000-U, Nikon Corp., Tokyo, Japan). The 3-D images of *E*. *coli* colonies were obtained from both instruments and then projected to 8-bit grayscale images, as shown in Fig 5. The projected images were compared for the evaluation based on the image quality. Fig 5A was taken with the custom-built confocal module equipped with a 20× objective lens with NA of 0.5 (UPlanFl 20×, Olympus Corp., Tokyo, Japan) whereas Fig 5B was measured with a 10× objective lens with an NA of 0.5 (CFI Super Fluor 10×, Nikon Corp., Tokyo, Japan). Because the 10× objective lens equipped on the proposed instrument had a low NA of 0.3, causing poor illustration of the 3-D morphology map, an objective lens with a higher numerical aperture was implemented for the measurement; a 20× objective lens with an NA of 0.5 was mainly used for the 3-D morphology map. In terms of resolution, Fig 5A has 128 × 128 pixels in the image, whereas Fig 5B has 512 × 512 pixels. Since the magnifications were different, FOVs were also different, which were 600 × 600 microns and 1208.32 × 1208.32 microns for Fig 5A and 5B, respectively. Unlike the confocal microscope, the bright-field microscope had a smaller FOV because of the small active area of the imaging sensor (See S5 Fig). With the given technical specifications, the custom-built confocal module could not illustrate colony morphology as detailed as the conventional confocal microscope. Nonetheless, both images well illustrated the outline of the colonies as well as the center elevation. In Fig 5C and 5D, the horizontal cross-section at the center of colonies is displayed for detailed comparison. Based on the reflectance data from the confocal images, Fig 5D looks better at illustrating the colony profile, but still, Fig 5C and 5D both showed that the fitted curve resembled Gaussian profile.

**Fig 6:**
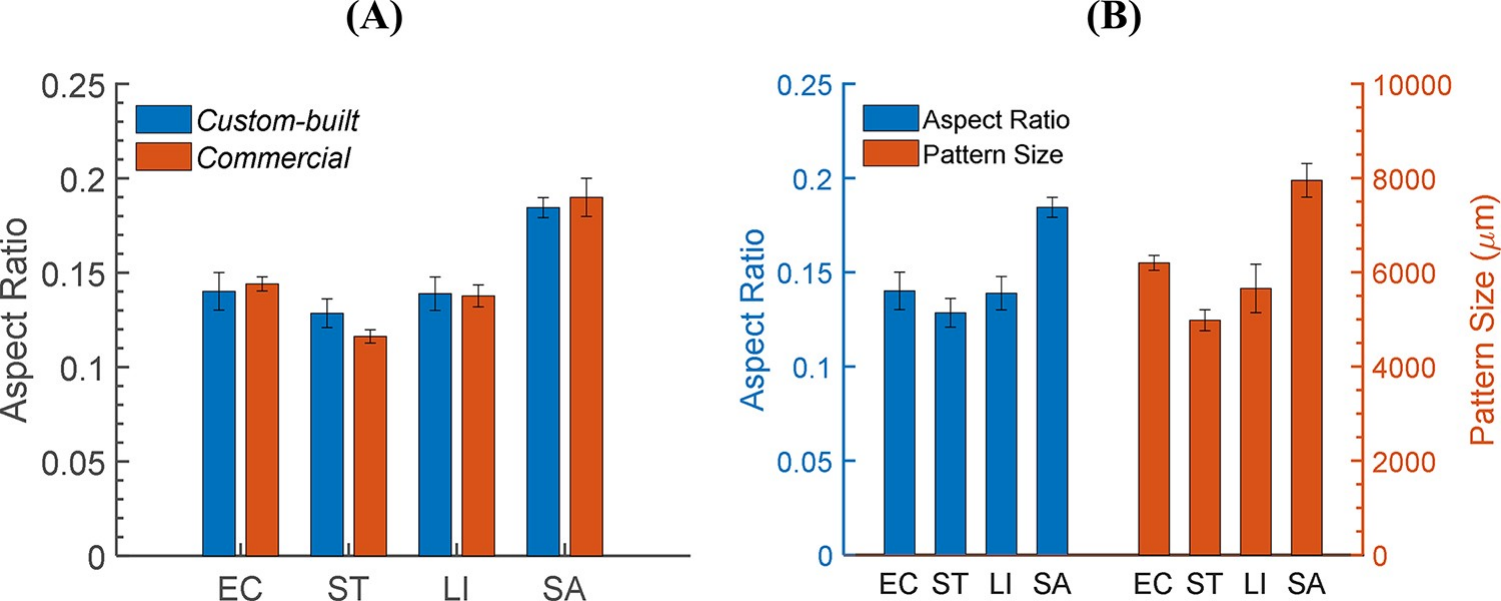
Precision of the aspect ratio and correlation to the pattern size. The performance of the custom-built confocal module was evaluated (A) by comparing the aspect ratios measured by the proposed instrument and by a confocal displacement meter. The calculated aspect ratio was compared to the size of the light-scatter pattern to prove (B) the relationship between the aspect ratio and the size of the scattering pattern (n = 10).

The aspect ratio of the colony, the ratio of center elevation to diameter, was an important parameter that could be obtained from a 3-D morphology map. Since the aspect ratio represented the overall slope of the colony, which showed a strong relationship to the diffraction angle and the pattern size, it needed to be accurately measured to associate with the light-scatter patterns. Therefore, the proposed instrument was once more challenged against another device, a confocal displacement meter (LT9010M, Keyence Corp., Osaka, Japan). For this evaluation, the diameters and heights of 10 selected colonies per genus were measured by both instruments. Because of the low resolution and manual z-stacking, in general, the center elevations measured on the multi-channel device were, on average, about 30.49% greater while the diameter was approximately 11.40% smaller. Thus, the diameter and height were calibrated based on these scales, and then the aspect ratio was computed. The average aspect ratios acquired from the 3-D morphology maps were 0.140, 0.129, 0.138, and 0.185 for *E*. *coli*, *S*. Typhimurium, *L*. *innocua*, *and S*. *aureus*, respectively. In Fig 6A, these aspect ratios were compared to those measured by the confocal displacement meter. Both methods showed that *S*. *aureus* formed colonies with the highest aspect ratio, followed by *E*. *coli*, *L*. *innocua*, and *S*. Typhimurium. However, aside from *S*. *aureus*, the aspect ratios measured on the multi-channel device were all at a similar level with no significant difference, as their error bars overlapped.

The correlation between the aspect ratio and the scattering pattern size has been extensively studied in previous reports, and it has been proved to be positive [8]. This relationship was once again confirmed in Fig 6B, as the aspect ratio and the pattern size of the four bacteria were plotted side by side for direct comparison. According to the aspect ratio, the pattern size of *S*. *aureus* and *S*. Typhimurium were nicely predicted to be the largest and the smallest. However, *E*. *coli* and *L*. *innocua* produced different sized patterns, although their aspect ratios were in a similar range. Nevertheless, the aspect ratio measured by the confocal displacement meter still nicely corresponds to the pattern size, which implies a strong correlation between aspect ratio and pattern size.

### Colony morphology and light-scatter pattern

**Fig 7:**
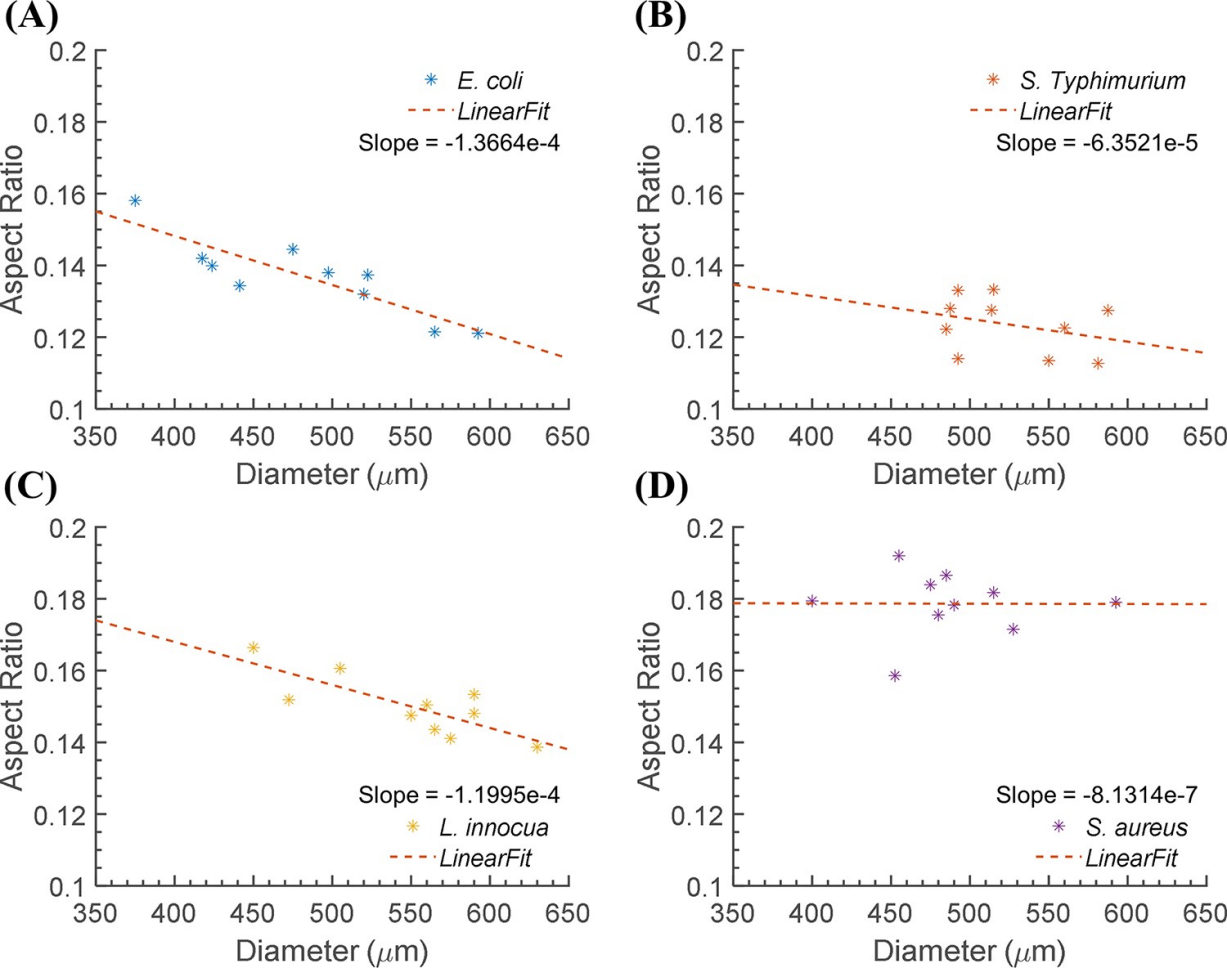
Comparison of the aspect ratio distribution for the four bacteria. Distribution of the aspect ratio of (A) *E*. *coli*, (B) *S*. Typhimurium, (C) *L*. *innocua*, and (D) *S*. *aureus*. The dashed lines represent the regression lines of the distribution (n = 10), and the slopes are given to illustrate the growth properties of each genus.

The calculated aspect ratios from the 3-D colony morphology maps were plotted against the diameter to investigate the growth properties of each bacterium on the TSA plate. Although the colonies of each bacterium were on the same plate and incubated for the same amount of time, the diameter and the height varied noticeably, resulting in large variations in the aspect ratio. The distribution of the aspect ratio was described using a least-square regression model, where the slope of the regression line indicates the ratio of growth in the lateral and vertical directions. Thus, a greater slope magnitude implies a faster growth rate for the diameter than for the height of the colony. In Fig 7, all the regression lines have negative slopes, meaning that the colonies grew faster in diameter than in height. However, the magnitude of the slope for the regression line of *S*. *aureus* was much smaller than for the other three by order of 2 to 3, showing that colonies of *S*. *aureus* grow much faster in the vertical direction.

**Fig 8:**
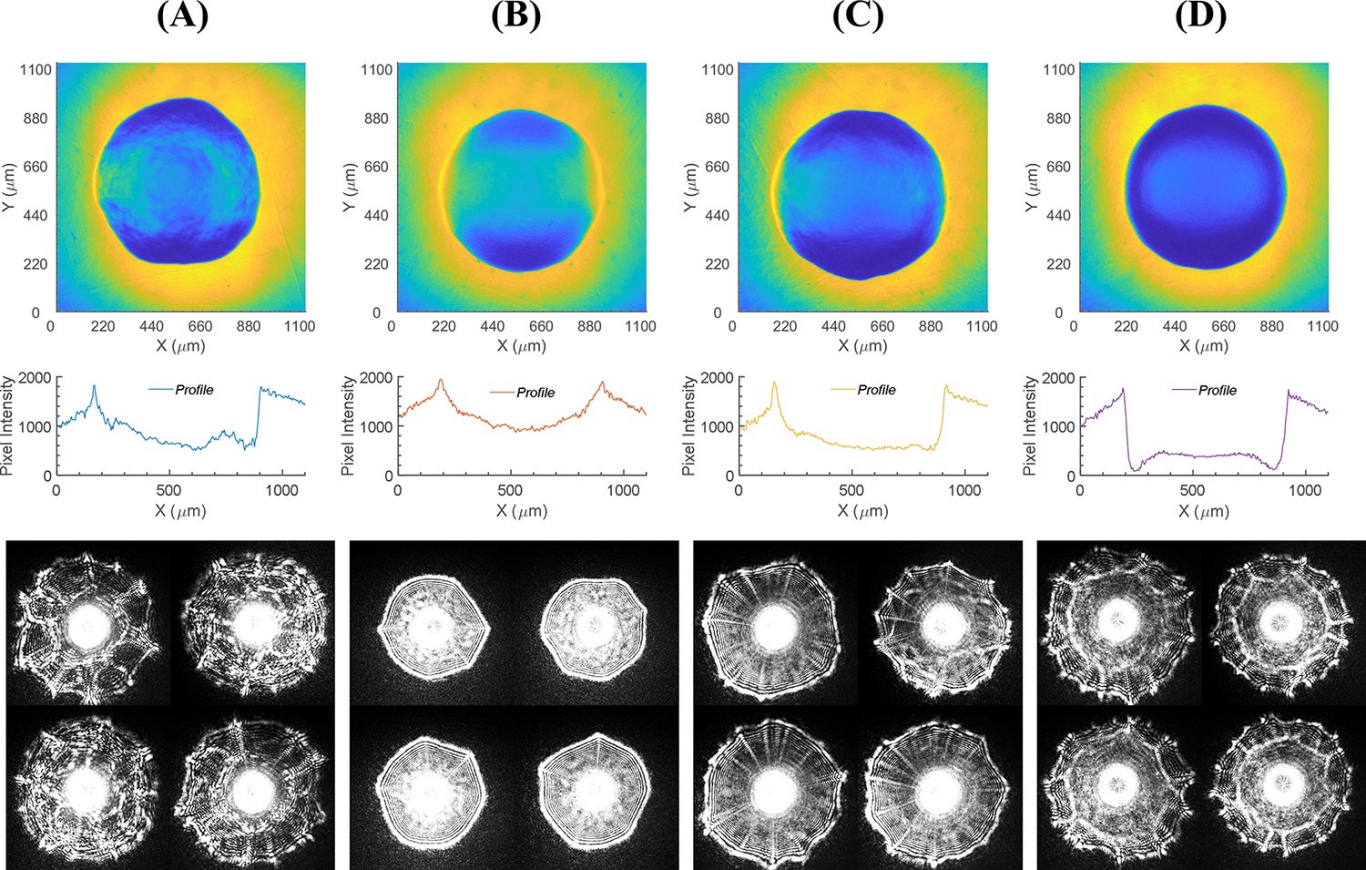
Effect of cold stress on colony morphology and light scatter pattern. Comparison of *E*.*coli* colony (A) before and (B) after cold incubation and *L*. *innocua* colony (C) before and (D) after cold incubation. Each row represents 2-D spatial OD distribution (top), cross-section profile across the center (middle), and light scatter patterns (bottom).

The morphological properties of a bacterial colony vary, depending not only on its species but also on environmental conditions such as nutrients, temperature, and its virulence or antimicrobial susceptibility. Therefore, an experiment was conducted to study the effect of temperature on the bacterial colony and eventually on the light-scatter pattern. When colony diameter reached 400–600 μm, the sample plates were stored in a refrigerator (~4 ˚C) for >48 hours. Interestingly, the optical properties of *E*. *coli* and *L*. *innocua* were noticeably changed, although the colony size remained nearly the same. To point out the difference in colonies before and after cold incubation, representative spatial OD maps, corresponding cross-section profiles, and the light-scatter patterns are presented in Fig 8. For *E*. *coli* (see Fig 8A and 8B), spatial OD was unevenly distributed throughout the colony, but after cold incubation, the distribution became uniform without any bumpiness. The cross-section profile also depicted an irregular distribution in OD that disappeared after cold incubation, as the profile became relatively smoother. For *L*. *innocua* (see Fig 8C and 8D), the spatial OD maps showed that the outer region of the colony became more optically dense, resulting in lower pixel intensity on each side of the colony edge in the cross-section profile. In terms of overall colony transparency, *E*. *coli* became more transparent, while *L*. *innocua* became more opaque. The comparison of the average pixel intensities, given in S6 Fig, shows that the average pixel intensity of the spatial OD map increased for *E*. *coli* but decreased for *L*. *innocua*. The change of optical properties in the colonies also significantly affected the shape of the scattering pattern. For example, the original scattering patterns of *E*. *coli* and *L*. *innocua* displayed ring patterns along with dominant web-like random interference, expressed as radial spoke pattern. After cold incubation, the scattering patterns of *E*. *coli* lost the spoke pattern; only the concentric rings remained. The pattern also became brighter owing to the change in transparency. *L*. *innocua* preserved the general structure of the scattering pattern, but a thick band formed inside the pattern, dividing it into inner and outer zones. The outer part of the pattern maintained a similar shape with the comb and spoke pattern, whereas the inner area became blurry.

## 4. Discussion

### Morphological and optical properties of bacteria colonies

The microscopic analysis demonstrated that *S*. *aureus* generally maintains a higher degree of circular symmetry than the other bacteria in terms of colony form. The reason is that individual *S*. *aureus* bacteria have a spherical shape with uniform arrangement during growth, whereas the others have rod-shaped cells that are randomly organized in the colony [16–18]. Another distinctive growth property of *S*. *aureus* clearly observed in the 3-D morphology maps was a higher aspect ratio; the elevation of an *S*. *aureus* colony was always higher than that of other colonies having a similar diameter range. In addition, the aspect ratio did not change significantly along the diameter as the vertical growth of an *S*. *aureus* colony was as fast as the lateral expansion. While the individual cell shape has a significant impact on the volume fill ratio for sphere and rod, the aspect ratio of a colony also indicates different local growth environments such as nutrition and oxygen availability from the agar. As cells move away from the agar surface, oxygen and nutrition diffusions become less effective compared to diffusion as the colony spreads horizontally. ELS methods can reveal this morphological information non-invasively by simply interrogating the whole volume of the colony with an incoming laser. Like the *S*. *aureus* colony form, the light-scatter patterns also maintained a higher degree of circular symmetry than did the other bacteria, and the steeper slope of the colony profile ultimately produced wider scattering patterns, since the maximum diffraction angle that determined the pattern size was governed by the magnitude of the maximum slope of the colony profile [9]. Previously, it was also reported that the scattering pattern was able to detect the morphological changes induced from not only the species but also the concentration of the agar and nutrition [7]. These studies imply that the colony profile is a crucial parameter in determining the shape of the scattering pattern since the amplitude and phase changes of the incident laser beam as it transmits through the colony are highly dependent on the 3-D colony structure.

To survive when exposed to environmental stresses, bacteria exhibit morphological changes at the cellular as well as the colony level. These changes are closely connected to the light-scatter patterns; the previous exploration of the correlations has proved that the media and the incubation temperature have a noticeable impact on colony morphology and diffraction pattern [7,19]. Fig 8 confirms that the incubation temperature significantly affected the colony structure of *E*. *coli* and *L*. *innocua*, and indeed, the shape of the light-scatter pattern. For *E*. *coli* colonies, the spatial OD turned into a smooth profile with decreased overall OD, leading to brighter, annular scattering patterns. This optical phenotype can be correlated with the biological effect during temperature changes. Since the *E*. *coli* colony typically consists of rod-shaped cells developing in somewhat irregular random patterns, the distribution of OD was not smooth throughout the colony. Therefore, a randomly arranged interference pattern with spokes was expected. However, the cold stress affected the composition of bacterial extracellular materials [20], so the absorption properties of the colony and subsequently, the shape of the scattering pattern was changed. Cold stress also significantly affected *L*. *innocua*, whose colony was divided into two sections with different ODs–center and peripheral area. Typically, in the center area, the oldest cells are located with a higher concentration of extracellular materials, developing higher mass density in the center of the colony [19]. Thus, OD distribution in this region was expected to be higher than in the region near the colony edge. However, the reverse was the case; the outer part became more optically dense than the center area. This fact was also demonstrated by a thick band that divided the light-scatter pattern into two sections, like the spatial OD map. This band formed because the colony was having a rapid change of OD in the radial direction, indicating a change of refractive index. Owing to transient growth arrest caused by a sudden drop in temperature, the size of the colonies did not change significantly even though the optical properties showed apparent transformation.

### Impact of the multi-channel instrument

In contrast to single-channel analysis, exploiting several channels in the analysis procedure enables better performance. For example, the imaging technique is one of the technologies that successfully implemented the idea of combining multiple modalities. By virtue of recent development in multimodal imaging probes, multimodal fluorescence imaging integrated with other imaging techniques, such as CT, PAI, PET, and MRI, has become a powerful tool in clinical research for improved detection sensitivity and accuracy [21,22]. Also, multi-channel approaches are popularly often found in nonlinear optical microscopy [11,23], confocal microscopy [24], and microspectroscopy [25]. These studies have proved that combining two or more techniques is a feasible approach to overcome the intrinsic limitations of a single modality analysis and have shown that the method is beneficial for additional comparative evaluation among multiple modalities. Therefore, a growing interest in bacterial discrimination using the light diffraction pattern also led to the development of optical multi-channel interrogation systems to thoroughly understand the ELS phenomenon and ultimately increase the sensitivity of ELS-based bacterial discrimination. *Buzalewicz et al*. first reported a multi-channel system that provided 2D distribution of transmission coefficients of bacterial colonies along with the Fresnel diffraction patterns [14]. The author recently integrated additional modalities, which were interferometric profilometry and spectral imaging system, for multi-parametric optical phenotyping and characterization of bacterial colonies [26]. This system shares similarities with the proposed instrument in that they both provide optical and morphological characteristics of the examined bacterial colony as additional information to the diffraction pattern; These systems were meant to measure the multi-spectral diffraction patterns, colony profiles, and spatial absorption properties of bacterial colonies. However, the methods utilized to measure those parameters were completely different. Unlike the system given in *Buzalewicz et al*., the proposed instrument integrated a confocal on a commercial microscope to measure the colony profile and spatial OD. Although the raster motion requires more time to measure a higher resolution image, the morphological and optical properties of the colony can be measured simultaneously in a single interrogation. Thanks to the design concept utilizing a commercial microscope for the instrument base, the proposed instrument has so much potential for expandability. With simple replacement of the objective lens, various sizes of bacterial colonies could be analyzed, and accordingly, a higher magnification could be achieved to interrogate bacteria at the cellular level. The integration of the infinite-corrected microscope could also benefit other microscopic approaches such as modalities that rely on fluorescence.

Utilizing the proposed instrument and following the operational procedure, coordinate-matched five different measurement channels were recorded for a bacterial colony without moving the specimen. The overall measurement time per colony was not more than six minutes, including time for mode switching. Since the proposed instrument was a prototype, mode switching was done manually and consumed most of the measurement time; the actual measurement time was less than 2.5 minutes. Therefore, measurement time could be dramatically reduced when an automation system is adopted for the next-generation instrument. The most time-consuming modality was the 3-D morphology map, which took about two minutes to collect and stack multiple images from confocal microscopy. Although a 128 × 128 raster takes only one second to completely scan the colony, owing to asynchronous serial communication (UART) between MCU and PC, there was a short wait time after scanning for complete data transfer. Utilizing synchronous communication instead of UART, such as SPI communication, could significantly reduce the wait time, as well as reduce the measurement time for 256 × 256. Eventually, 3-D morphology maps with higher resolution can be measured in much less time.

The image quality of the 3D morphology and spatial OD maps need to be improved. For example, in Figs 3 and 8, the spatial OD maps have uneven intensity distributions where the center is much brighter than the corners of the images. Moreover, the intensity distributions within the colonies were not perfect symmetry although their forms were correctly measured. These issues were caused by the raster mechanism of the confocal laser scanning microscope. The 2-axis galvanometer refracts incoming light to scan a plane field although the laser beam is focused on a spherical surface. Therefore, the focal plane is not perfectly flat, and the corners of the images were appeared to be darker than the center. This could be fixed by the implementation of correcting lenses or by calibration based on a reference image since the uneven intensity variation is consistent throughout the measurement.

## Supporting information

S1 Fig Pictures of the multi-channel instrument. (A) The optical multi-channel interrogation instrument; (B) a custom-built confocal laser module attached to the upright microscope; (C) the main controller unit of the system.
(DOCX)

S2 Fig Circuit diagram of a multi-purpose pre-amp board. The custom-built PCB board for the multi-purpose pre-amp was designed to have an I/V converter, two inverting voltage amplifiers, and a 2^nd^-order low-pass filter to process raw signals from photodiodes and galvanometer.
(DOCX)

S3 Fig 2-D spatial OD distributions of sample bacteria and average pixel intensities. (A) Representative images of 2-D spatial OD distribution for the four bacteria types, measured with a 10× objective lens. Backgrounds were removed to calculate the average pixel intensities of the colonies. (B) Corresponding average pixel intensities are displayed for comparison (n = 10).
(DOCX)

S4 Fig Change of light-scatter pattern with respect to the incident wavelength. The cross-sectional view of the spectral light scatter patterns of (A) *L*. *innocua* and (B) *S*. *aureus* are presented where the local maxima represent the rings in the scattering pattern images. The comparison clearly shows that, as the wavelength increased, the number and thickness of the ring decreased. For *L*. *innocua*, the number of rings decreased from 24< to 19 to 13 for and from 27< to 25 to 17 for *S*. *aureus*. Although it was difficult to count the rings for 405 nm because of the noises in the pattern images, they were more than 635 and 904 nm. The location of the most outer ring moved from 397 to 373 to 357 pixel for *L*. *innocua*, and from 438 to 422 to 403 for *S*. *aureus*.
(DOCX)

S5 Fig Microscopic images of a calibration slide. A microscopic calibration slide with a 1-mm scale/0.01-mm division was tested under the proposed instrument with a 20× objective lens. Fields of view (FOVs) illustrated by (A) bright-field microscopy, (B) confocal microscopy for 3-D morphology map, and (c) 2-D spatial OD map. The field of view for the bright-field microscope was smaller than the others because of the small active area of the imaging sensor.
(DOCX)

S6 Fig Morphological analysis of bacteria colonies incubated at a cold temperature. Bacteria colonies incubated at a cold temperature were analyzed by 3-D morphology and 2-D spatial OD maps to compare their morphological properties to those of normally incubated colonies. (A) Change in aspect ratio after cold incubation (n = 10). (B) Change in transparency after cold incubation, given as the average pixel intensity of the 2-D spatial OD map (n = 10).
(DOCX)
