# Supplementary material for: Optical multi-channel interrogation instrument for bacterial colony characterization
Source: PLoS One. 2021 Feb 25;16(2):e0247721. doi: 10.1371/journal.pone.0247721 (PMC7906345; doi:10.1371/journal.pone.0247721)
Supplement: S1 Fig — (A) The optical multi-channel interrogation instrument; (B) a custom-built confocal laser module attached to the upright microscope; (C) the main controller unit of the system. (DOCX) [file pone.0247721.s001.docx]

Optical Multi-channel Interrogation Instrument for Bacterial Colony Characterization

Supporting information

Iyll-Joon Doh^a^, Huisung Kim^a^, Bartek Rajwa^d^, J. Paul Robinson^b,c^, and Euiwon Bae^a^

^a^Applied Optics Laboratory, School of Mechanical Engineering; ^b^Weldon School of Biomedical Engineering; ^c^Basic Medical Sciences, College of Veterinary Medicine; ^d^Bindley Bioscience Center, Purdue University, West Lafayette, IN 47907, USA

Corresponding author: Euiwon Bae (ebae@purdue.edu)


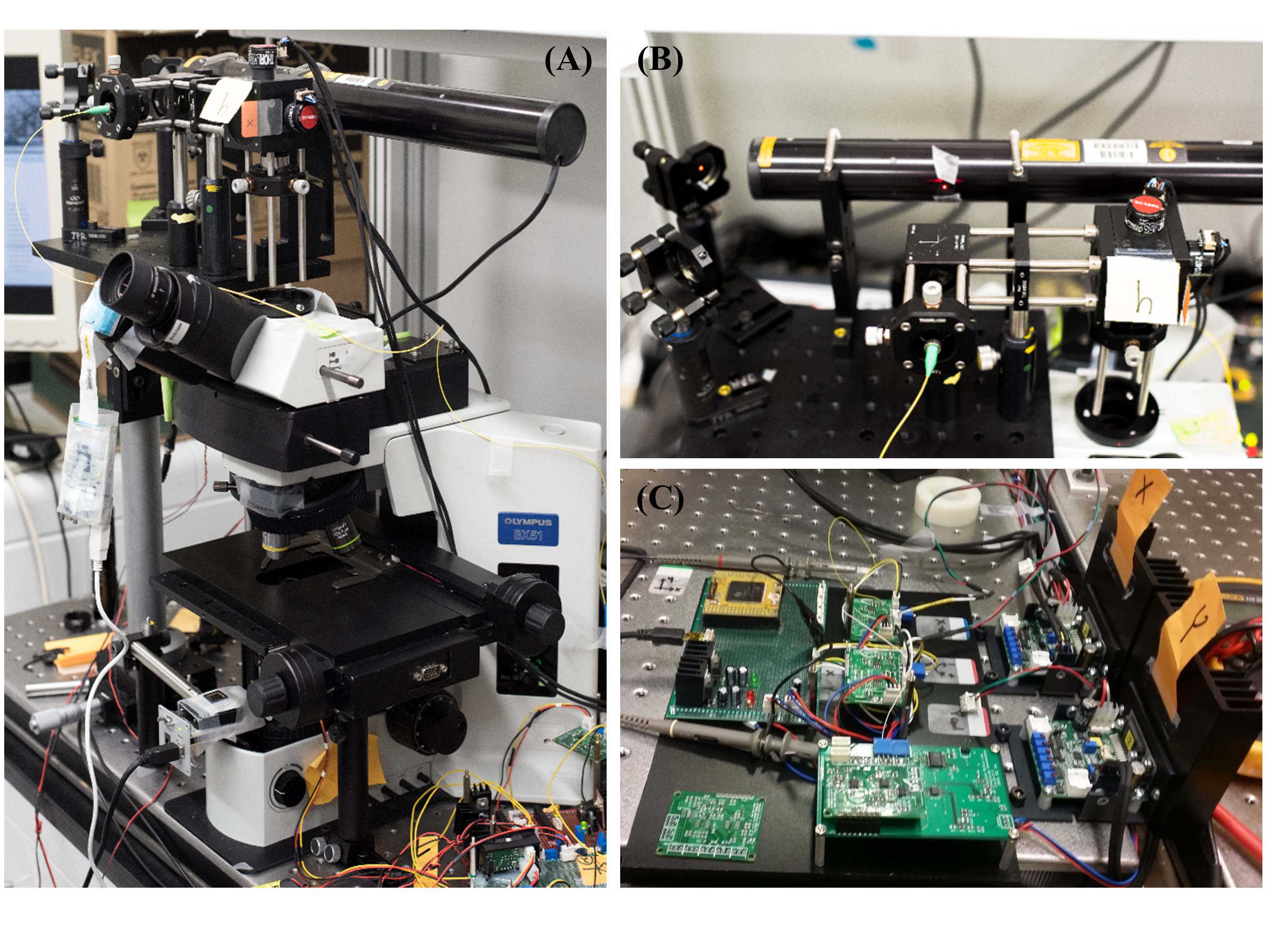


**Figure S1. Pictures of the multi-channel instrument.**

(A) The optical multi-channel interrogation instrument; (B) a custom-built confocal laser module attached to the upright microscope; (C) the main controller unit of the system.
