# Supplementary material for: Optical multi-channel interrogation instrument for bacterial colony characterization
Source: PLoS One. 2021 Feb 25;16(2):e0247721. doi: 10.1371/journal.pone.0247721 (PMC7906345; doi:10.1371/journal.pone.0247721)
Supplement: S2 Fig — The custom-built PCB board for the multi-purpose pre-amp was designed to have an I/V converter, two inverting voltage amplifiers, and a 2nd-order low-pass filter to process raw signals from photodiodes and galvanometer. (DOCX) [file pone.0247721.s002.docx]

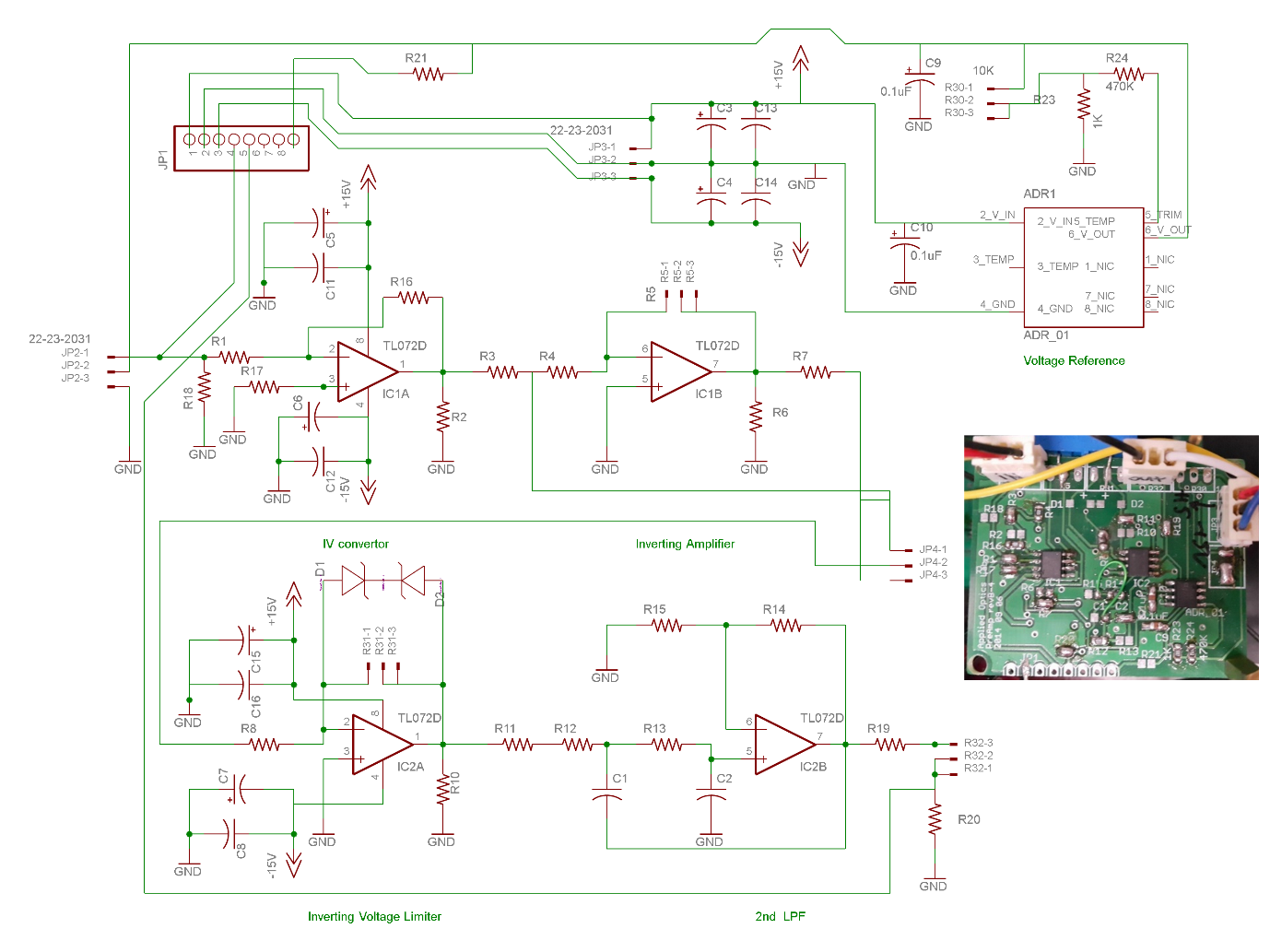


**Figure S2. Circuit diagram of a multi-purpose pre-amp board.**

The custom-built PCB board for the multi-purpose pre-amp was designed to have an I/V converter, two inverting voltage amplifiers, and a 2^nd^-order low-pass filter to process raw signals from photodiodes and galvanometer.
