# Supplementary material for: Optical multi-channel interrogation instrument for bacterial colony characterization
Source: PLoS One. 2021 Feb 25;16(2):e0247721. doi: 10.1371/journal.pone.0247721 (PMC7906345; doi:10.1371/journal.pone.0247721)
Supplement: S3 Fig — (A) Representative images of 2-D spatial OD distribution for the four bacteria types, measured with a 10× objective lens. Backgrounds were removed to calculate the average pixel intensities of the colonies. (B) Corresponding average pixel intensities are displayed for comparison (n = 10). (DOCX) [file pone.0247721.s003.docx]

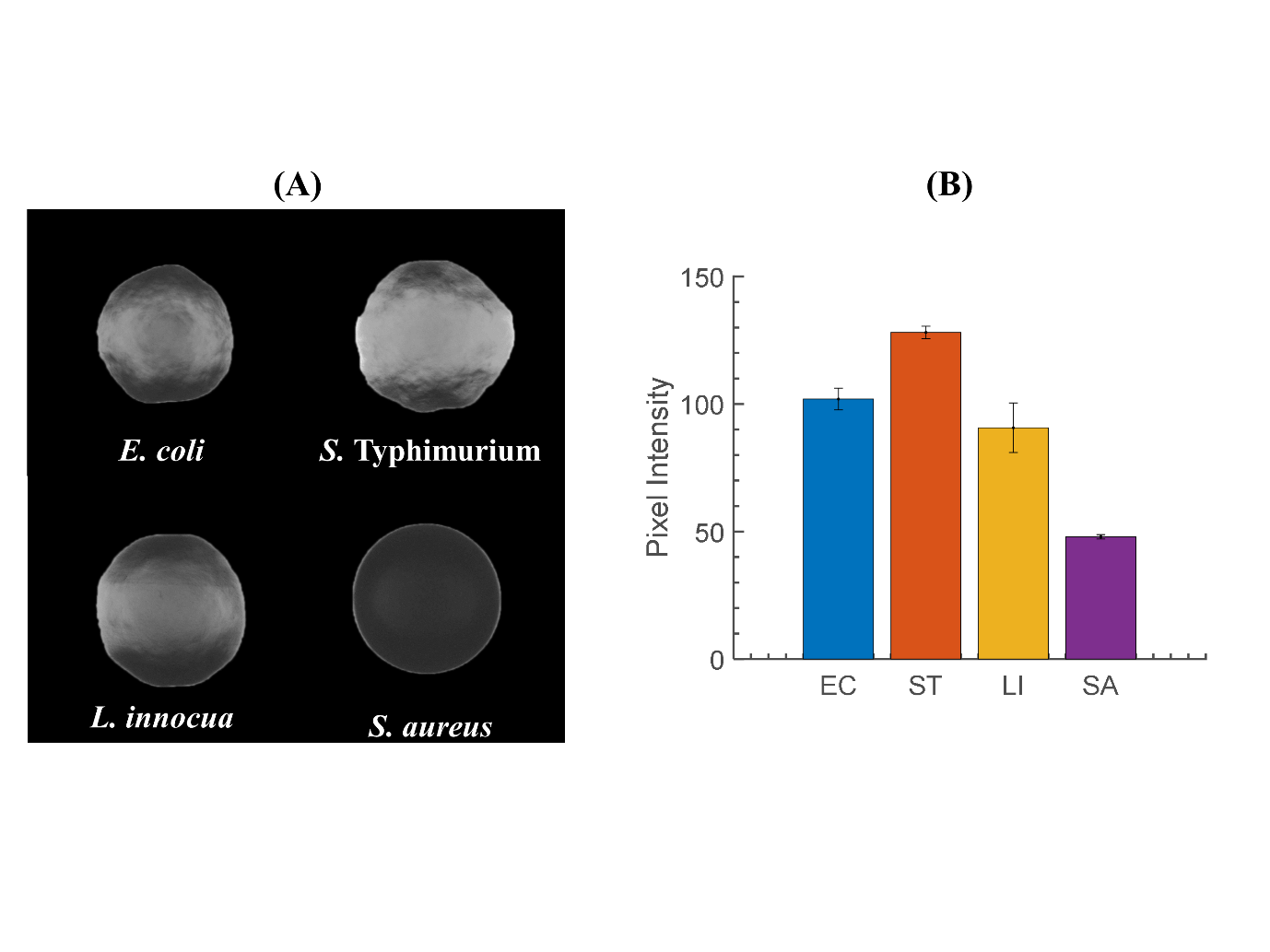


**Figure S3. 2-D spatial OD distributions of sample bacteria and average pixel intensities.**

(A) Representative images of 2-D spatial OD distribution for the four bacteria types, measured with a 10× objective lens. Backgrounds were removed to calculate the average pixel intensities of the colonies. (B) Corresponding average pixel intensities are displayed for comparison (n = 10).
