# Supplementary material for: Optical multi-channel interrogation instrument for bacterial colony characterization
Source: PLoS One. 2021 Feb 25;16(2):e0247721. doi: 10.1371/journal.pone.0247721 (PMC7906345; doi:10.1371/journal.pone.0247721)
Supplement: S4 Fig — The cross-sectional view of the spectral light scatter patterns of (A) L. innocua and (B) S. aureus are presented where the local maxima represent the rings in the scattering pattern images. The comparison clearly shows that, as the wavelength increased, the number and thickness of the ring decreased. For L. innocua, the number of rings decreased from 24< to 19 to 13 for and from 27< to 25 to 17 for S. aureus. Although it was difficult to count the rings for 405 nm because of the noises in the pattern images, they were more than 635 and 904 nm. The location of the most outer ring moved from 397 to 373 to 357 pixel for L. innocua, and from 438 to 422 to 403 for S. aureus. (DOCX) [file pone.0247721.s004.docx]

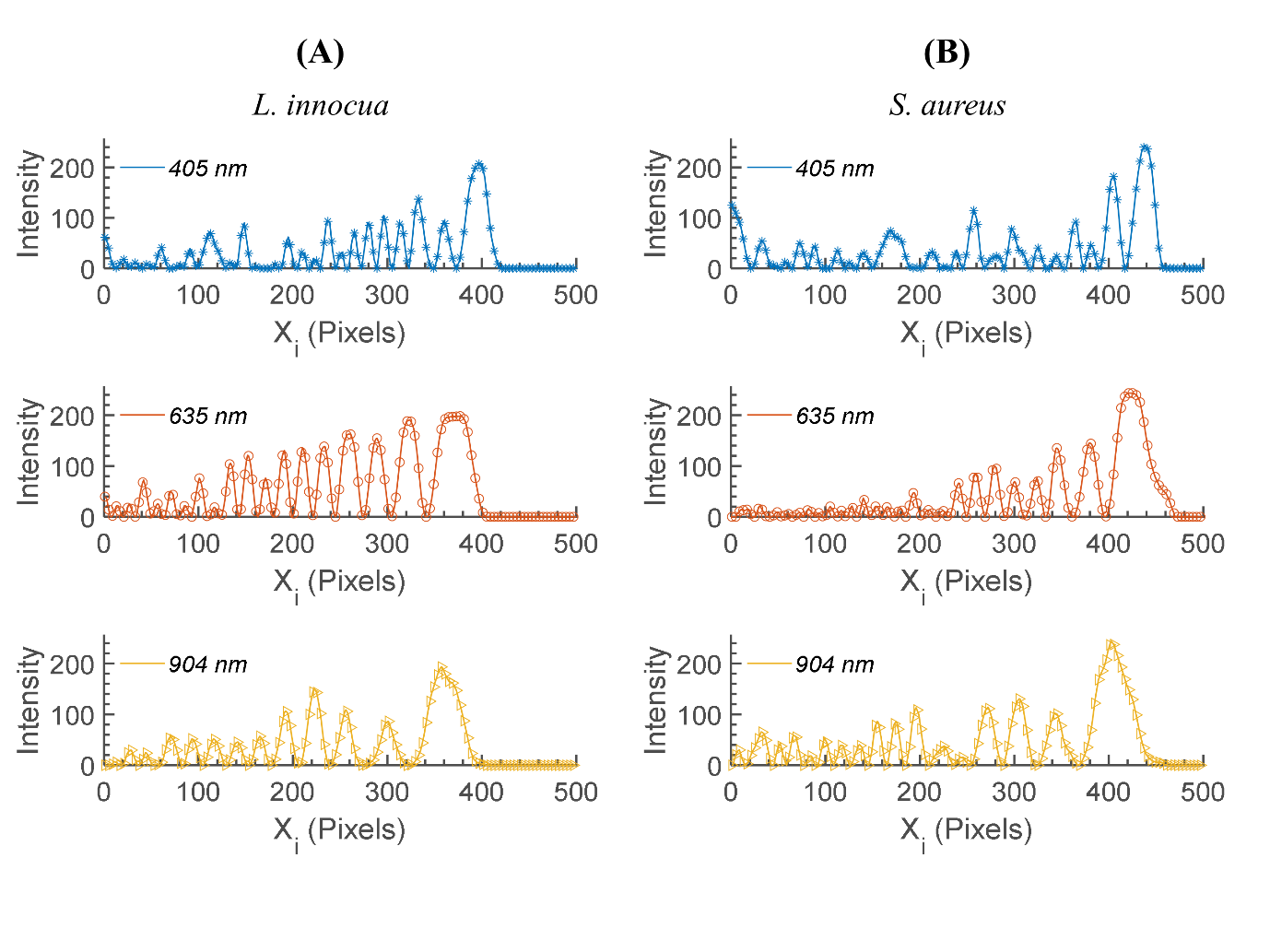


**Figure S4. Change of light-scatter pattern with respect to the incident wavelength**

The cross-sectional view of the spectral light scatter patterns of (A) *L. innocua* and (B) *S. aureus* are presented where the local maxima represent the rings in the scattering pattern images. The comparison clearly shows that, as the wavelength increased, the number and thickness of the ring decreased. For *L. innocua*, the number of rings decreased from 24< to 19 to 13 for and from 27< to 25 to 17 for *S. aureus*. Although it was difficult to count the rings for 405 nm because of the noises in the pattern images, they were clearly more than 635 and 904 nm. The location of the most outer ring moved from 397 to 373 to 357 pixel for *L. innocua*, and from 438 to 422 to 403 for *S. aureus*.
