# Supplementary material for: Optical multi-channel interrogation instrument for bacterial colony characterization
Source: PLoS One. 2021 Feb 25;16(2):e0247721. doi: 10.1371/journal.pone.0247721 (PMC7906345; doi:10.1371/journal.pone.0247721)
Supplement: S5 Fig — A microscopic calibration slide with a 1-mm scale/0.01-mm division was tested under the proposed instrument with a 20× objective lens. Fields of view (FOVs) illustrated by (A) bright-field microscopy, (B) confocal microscopy for 3-D morphology map, and (c) 2-D spatial OD map. The field of view for the bright-field microscope was smaller than the others because of the small active area of the imaging sensor. (DOCX) [file pone.0247721.s005.docx]

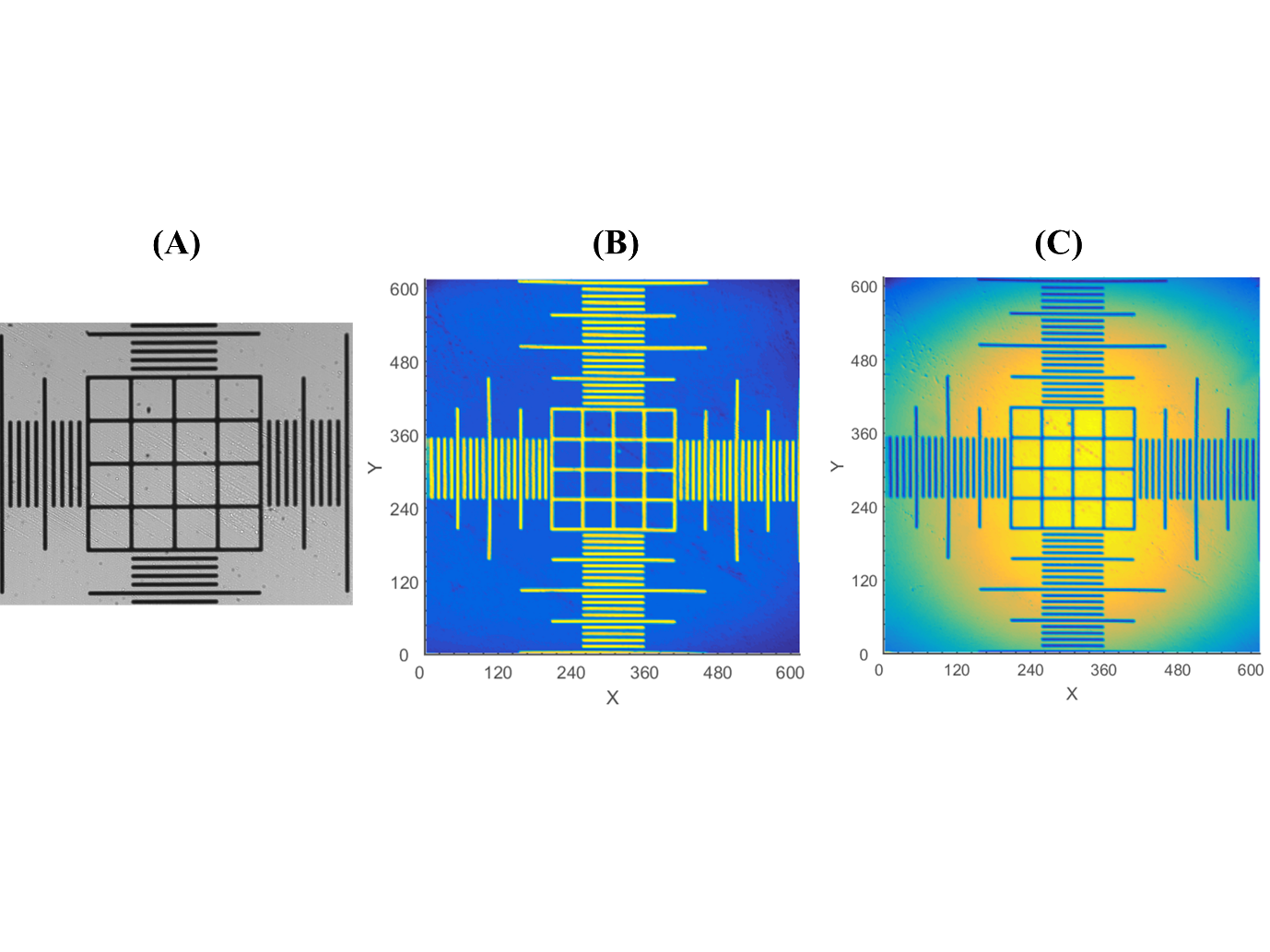


**Figure S5. Microscopic images of a calibration slide.**

A microscopic calibration slide with a 1-mm scale/0.01-mm division was tested under the proposed instrument with a 20× objective lens. Fields of view (FOVs) illustrated by (A) bright-field microscopy, (B) confocal microscopy for 3-D morphology map, and (c) 2-D spatial OD map. The field of view for the bright-field microscope was smaller than the others because of the small active area of the imaging sensor.
