# Supplementary material for: Optical multi-channel interrogation instrument for bacterial colony characterization
Source: PLoS One. 2021 Feb 25;16(2):e0247721. doi: 10.1371/journal.pone.0247721 (PMC7906345; doi:10.1371/journal.pone.0247721)
Supplement: S6 Fig — Bacteria colonies incubated at a cold temperature were analyzed by 3-D morphology and 2-D spatial OD maps to compare their morphological properties to those of normally incubated colonies. (A) Change in aspect ratio after cold incubation (n = 10). (B) Change in transparency after cold incubation, given as the average pixel intensity of the 2-D spatial OD map (n = 10). (DOCX) [file pone.0247721.s006.docx]

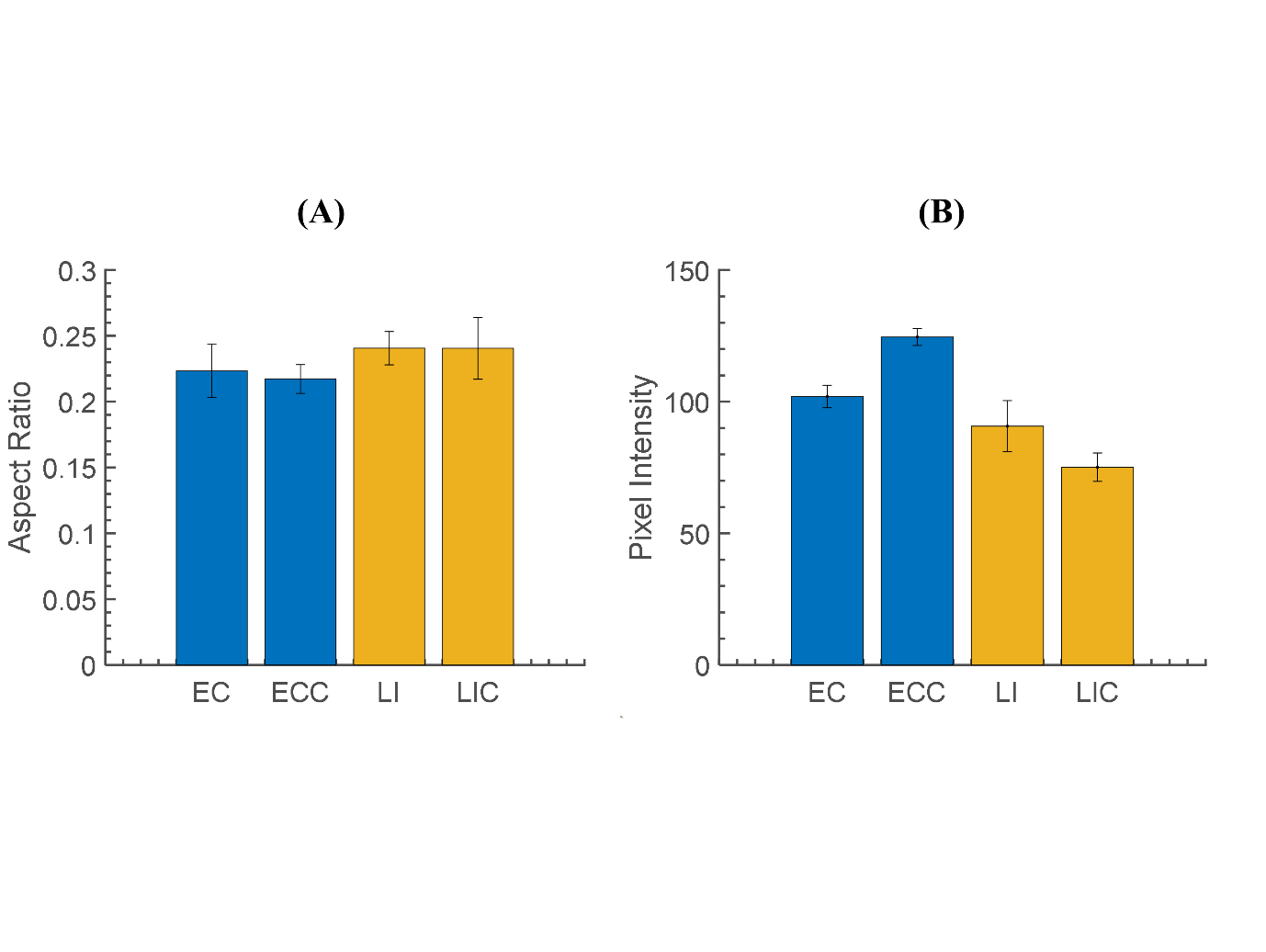


**Figure S6. Morphological analysis of bacteria colonies incubated at a cold temperature.**

Bacteria colonies incubated at a cold temperature were analyzed by 3-D morphology and 2-D spatial OD maps to compare their morphological properties to those of normally incubated colonies. (A) Change in aspect ratio after cold incubation (n = 10). (B) Change in transparency after cold incubation, given as the average pixel intensity of the 2-D spatial OD map (n = 10).
